# Comparative efficacy and safety of topical caspofungin 0.5% and voriconazole 1% for fungal keratitis: a pilot clinical trial

**DOI:** 10.1038/s41598-025-22615-w

**Published:** 2025-11-06

**Authors:** Mehrnaz Atighehchian, Golshan Latifi, Ahmed Shalaby Bardan, Dean Ouano, Siamak Zarei-Ghanavati, Zohreh Abedinifar, Mehran Zarei-Ghanavati

**Affiliations:** 1 Farabi Eye Hospital, Tehran University of Medical Sciences, Tehran, Iran; 2 Department of Ophthalmology, UT Health San Antonio, San Antonio, TX USA; 3 Ophthalmology department, Faculty of Medicine, Alexandria University, Alexandria, Egypt; 4 Leeds Teaching Hospitals, NHS Trust, Leeds, UK; 5 Coastal Eye Clinic, New Bern, NC USA; 6 Eye Research Center, Mashhad University of Medical Sciences, Mashhad, Iran; 7 Microbiology Unit, Department of Pathology, Farabi Hospital, Tehran University of Medical Sciences, Tehran, Iran

**Keywords:** Fungal keratitis, Topical, Voriconazole, Caspofungin, Fusarium, Drug discovery, Microbiology, Diseases

## Abstract

To compare the efficacy of topical caspofungin 0.5% with topical voriconazole 1% in patients with fungal keratitis. In this pilot clinical trial, thirty-four eyes from 34 patients diagnosed with fungal keratitis, confirmed by positive smear or culture results or in vivo confocal microscopy (IVCM), were included in the study. Patients were randomized to receive either topical caspofungin 0.5% or voriconazole 1%. The primary outcome measure was best spectacle-corrected visual acuity (BSCVA) at 3 months of follow-up. Secondary outcomes were the percentage of healed ulcers, time to healing, and scar size. After 3 months of treatment, the mean BSCVA was1.17 ± 0.85 logarithm of minimum angle of resolution (LogMAR) units in the caspofungin group and 0.49 ± 0.56 LogMAR units in the voriconazole group and the difference between two groups was 0.18 LogMAR (95% CI, -0.14 to 0.51 P = 0.276). The final mean scar size was similar between the caspofungin group (2.47 ± 2.1) and the voriconazole group (3.09 ± 1.11) (P = 0.254, 95% CI, -1.88 to 0.50 mm). The percentage of cases healed was 70.6% (12/17) in the caspofungin group and 100% (17/17) in the voriconazole group (P = 0.060, Hazard ratio ,2.06 95% CI,0.97 to 4.37). Fusarium species were the most common species (9/34; 26.5% cases). Complete stromal healing occurred in all 5 patients infected with Fusarium species who were treated with voriconazole 1%. one out of 4 patients in the caspofungin group showed complete healing (P = 0.048). The patients in caspofungin group had large mean infiltration size 4.81± 1.60 mm, (P = 0.255) than 2.90± 1.60 mm in voriconazole group and deeper infiltration depth 25 ±13% than 15± 9%voriconazole group (P = 0.120). This study indicates that topical caspofungin 0.5% may be effective as topical voriconazole 1%, making caspofungin 0.5% a viable primary treatment option for patients with fungal keratitis.

**Supplementary Information:**

The online version contains supplementary material available at 10.1038/s41598-025-22615-w.

## Introduction

Fungal keratitis (FK) is a significant cause of infectious keratitis in certain tropical regions^1–3^. Several antifungal medications are available, typically divided into two main categories: polyenes (such as natamycin and amphotericin-B) and azoles (including fluconazole and voriconazole)^2,4^. Natamycin is the preferred treatment choice for filamentous FK due to its excellent tolerability, stability, and broad-spectrum efficacy^3,4^. However, natamycin poses some clinical challenges, including its high price, limited global availability and poor corneal penetration caused by its high molecular weight^4^. Topical voriconazole has also been shown to be effective in treating FK, whether used alone or in combination with systemic voriconazole^1^.

Given the ongoing challenges regarding the availability of effective antifungal drops, it is crucial to investigate and assess alternative antifungal agents^5^. Echinocandins, the newest class of antifungal medications, were first introduced in 2001. These drugs block an enzyme pathway that is required for the synthesis of beta-(1–3)-D-glucan, a critical component of the fungal cell wall^6^. Caspofungin, an echinocandin, is an FDA-approved treatment for invasive candidiasis, refractory invasive aspergillosis, and the prophylactic management of febrile neutropenic patients^7–9^. Emerging case reports suggest that topical caspofungin 0.5% may be effective in managing FK^5,10–13^. While caspofungin has demonstrated significant in vitro activity against a range of fungal pathogens, its effectiveness against Fusarium is less consistent^14^. Despite its potential, no clinical trials have directly compared the efficacy of topical caspofungin with other antifungal agents for treating FK^11^. This pilot clinical trial is the first to evaluate and compare the safety and efficacy of topical caspofungin 0.5% with voriconazole 1% in the treatment of FK.

## Methods

The study was a double-blind pilot clinical trial conducted in accordance with the principles of the Declaration of Helsinki and was approved by the institutional ethics committee at Tehran University of Medical Sciences (IR.TUMS.FARABIH.REC.1402.027). Informed consent was obtained from all enrolled patients. We screened consecutive patients presenting to Farabi Eye Hospital, Tehran University of Medical Sciences, with unilateral FK between February to September 2024 for the following criteria. This clinical trial study was registered at irct.behdasht.gov.ir (IRCT20231219060471N1 16/02/2024).

Patients aged over 18 years with fungal corneal ulcers confirmed by smear and/or culture, or those diagnosed with (FK through in vivo corneal confocal microscopy (IVCM) in smear- or culture-negative cases, lesions exceeding 2 mm in size and involving up to 2/3rd of stroma with or without anterior chamber reaction and who were required to be willing to adhere to regular follow-up visits were included.

Complete medical history was obtained, including age, sex, occupation, symptoms, onset, and predisposing factors such as trauma, steroid use, or recent surgery. Patients were specifically asked about any systemic diseases, including diabetes mellitus. Baseline data, including best spectacle-corrected visual acuity (BSCVA) was measured with the LogMAR scale, were collected. Slit-lamp examinations and baseline photographs were taken, with and without fluorescein staining. The corneal ulcers were described in terms of the size of the epithelial defect and the infiltration with measurements taken along the longest axis and an axis perpendicular to it. The average size was calculated using the geometric mean of these two dimensions. The depth of infiltration was estimated by comparing it to the normal corneal thickness observed through the slit lamp. Additionally, hypopyon and cataract presence were noted. Ultrasonography was performed on patients whose posterior segments were not visible, to rule out endophthalmitis.

Corneal scrapings were taken from the base and edge of the infiltration under sterile conditions. Microbiological investigations included potassium hydroxide (KOH) wet mount preparation, gram smear, and cultures on blood agar, chocolate agar, and Sabouraud dextrose agar. In smear—or culture-negative cases, IVCM was performed to confirm diagnosis by identifying highly reflective, branching, bifurcating linear structures.

This study randomly assigned 34 eyes from 34 patients into two groups using a permuted block randomization method (random block length: 2, 4). Both the patients and the clinician evaluating the patients are masked. Each group consisted of 17 patients. Group 1 received topical caspofungin 0.5%, while group 2 was treated with topical voriconazole 1%. The study was reported in accordance with the (CONSORT) guidelines (Additional file 1).

For the first 48 h, both groups received their respective treatments every hour. After this initial period, the frequency was reduced to every 2 h until healing was achieved, followed by administration every 4 h for three weeks. Further tapering decision was based on the clinician’s judgment and the complete healing of the corneal ulcer. All patients were examined daily in the hospital until they were discharged and were followed up on days 7, 14, 30, and 3 months. Corneal ulcer debridement is considered based on the specific needs of each patient.

In the voriconazole group, if there was poor response by day 7 to 10, intrastromal voriconazole injections were considered in addition to oral voriconazole (200 mg twice daily). Liver function tests were routinely monitored during the treatment period.

In the caspofungin group, if there was a poor response by the 7th to 10th day of treatment, topical voriconazole 1% was added, following the same frequency schedule. Additionally, intrastromal voriconazole injections and oral voriconazole (200 mg twice daily) was initiated.

Poor response keratitis was characterized by an ulcer that showed no improvement, a decrease of less than 20% in stromal infiltrate, an increase in the size of the infiltration, or an increase in the size of the hypopyon by the seventh day.

During each visit, patients were evaluated for the size of the infiltration and epithelial defect, the extent and depth of infiltration, the presence and size of hypopyon, the formation and size of the corneal scar, the duration of corneal healing, and final visual acuity. Outcomes were compared between the two groups.

The scar size was measured with a slit lamp, recording its largest dimension along with the measurement taken along an axis perpendicular to it. Any complications and adverse effects (e.g., increased size and depth of infiltration, descemetoceles, corneal perforation, and endophthalmitis) were documented. Patients with poor response to treatment by three weeks were considered treatment failures and were scheduled for therapeutic keratoplasty.

### The dosage regimen for the topical caspofungin and voriconazole

Fortified eye drops were formulated in the Department of Ocular Pharmacology at Farabi Eye Hospital, Tehran University of Medical Sciences. All preparations were conducted under sterile conditions, and the sterility of fortified eye drops was confirmed prior to their administration to patients. For topical voriconazole 1%, 200 mg of voriconazole powder was reconstituted in 19 ml of Ringer’s lactate solution. For the preparation of topical caspofungin at a concentration of 0.5%, one vial containing 50 mg of caspofungin acetate is diluted in 10.5 mL of sterile normal saline. It is essential that all eye drop formulations are prepared freshly on a weekly basis and stored at a temperature of 4 °C while being protected from light exposure. The intra-stromal injection involved administering 50 mg (0.1 mL) of reconstituted voriconazole solution. This was done using a 1 mL tuberculin syringe fitted with a 30-gauge needle. The needle was inserted at an angle into the cornea, starting from the clear area and reaching the mid-stromal level of the ulcer. Multiple injections were then given around the ulcer.

### Statistical methods

We presented the data using various statistical measures, including mean, median, standard deviation, range, frequency, and percentage. Variables between the groups were compared using the t-test, Mann-Whitney test, Chi-square test, and Fisher’s exact test. We also used the Shapiro-Wilk test alongside Q-Q plots to assess the normal distribution of the data. To determine the relationship between variables and the intensity of certain events, we conducted Cox regression analysis, reporting the hazard ratio (HR) along with its corresponding 95% confidence interval (CI). To compare visual acuity and scar size between the two groups, we reported both unadjusted and adjusted mean differences using a general linear model. All statistical analyses were carried out using SPSS software (version 25.0). A P-value of less than 0.05 was considered statistically significant.

## Results

The baseline characteristics of the two groups were comparable (Table 1). The mean age of the patients was 55 ±11 and 48 ± 14 years in caspofungin and voriconazole groups, respectively (*P* = 0.121). Of the total number of patients, 26 (76.5%) were males, and eight were females (23.5%). A total of 22 patients (64.7%) reported a history of prior trauma. A total of 30 patients (88.23%) exhibited a positive result on the first day, while 4 patients were diagnosed with IVCM.

**Table 1:** Baseline parameters in cases of fungal keratitis

Parameter				Treatment			P-value
				Caspofungin	Voriconazole		
Demographic data Age (year)				55 ± 11	48 ± 14		0.121
Female (%)				7 (41.2)	1 (5.9)		0.039
Base-line BSCVA (LogMar)				1.72 ± 1.13	1.56 ± 1.13		0.602
Diabetes melilotus (%)				6 (35.3)	1 (5.9)		0.085
Trauma				12 (70.6)	10 (58.8)		0.721
Topical steroid usage (%)				0 (0.0)	2 (11.8)		0.485
Clinical data							
Depth of infiltration (%)				28 ± 11	22 ± 12		0.109
Mean size of stromal infiltration (mm)				4.25 ± 1.54	3.74 ± 1.23		0.500
Mean size of epithelial defect (mm)				4.34 ± 1.92	3.03 ± 1.7		0.061
Symptoms onset (days)				13 ± 7	10 ± 7		0.103
Ulcer with hypopyon (%)				6 (35.3)	3 (17.6)		0.438
Ulcers involving central 3-mm (%)				10 (58.8)	14 (82.4)		0.132
Endothelial plaque (%)				4 (23.5)	1 (5.9)		0.435
Microbiological data							
First-day positive smear (%)				15 (88.2)	15 (88.2)		>0.99
Sixth-day positive smear (%)				10 (66.7)	7 (46.7)		0.462

Among these, 12 out of 17 patients (70.6%) in the caspofungin group and 10 out of 17 patients (58.8%) in the voriconazole group sustained trauma involving vegetable matter. The mean time from symptom onset to presentation was 13 ± 7 days and 10 ± 7 days in the caspofungin and the voriconazole groups, respectively (*P* = 0.103). The average size of the ulcers measured along the longest axis and perpendicular to it was 4.25 ± 1.54 mm in the caspofungin group and 3.74 ±1.23 mm in the voriconazole group (*P* = 0.50*)*.

### Primary outcome

The BSCVA after 3 months of treatment improved from baseline in 13 of 17 patients (81.3%) in the caspofungin group and in 15 of 17 patients (88.2%) in the voriconazole group (*P* = 0.656). The mean final BSCVA after treatment that was adjusted based on age, size, and depth of ulcers was 1.17± 0.85 LogMAR units in the caspofungin group and 0.49 ±0.56 LogMAR units in the voriconazole group and difference between two groups was 0.18 LogMAR (95% CI, −0.14 to 0.51 *P* = 0.276). (Table 2).

**Table 2:** Change BSCVA *and Scar size ** in patients with fungal keratitis on topical Caspofungin 0.5% and topical voriconazole 1% therapy.

Variable	Treatment		Unadjusted				Adjusted			
			Difference	95% CI		P	Difference	95% CI		P
	Caspofungin	Voriconazole		Lower	Upper			Lower	Upper	
Baseline VA	1.72 ± 1.13	1.56 ± 1.13	0.16	-0.58	0.89	0.676	-			
Final VA	1.17 ± 0.85	0.49 ± 0.56	0.68	0.21	1.15	0.005	0.18	-0.14	0.51	0.276
VA change*	0.64 ± 1.18	1.07 ± 0.95	-0.43	-1.13	0.28	0.233	0.18	-0.14	0.51	0.276
Scar size**	2.47 ± 2.1	3.09 ± 1.11	-0.62	-1.72	0.48	0.270	-0.69	-1.88	0.50	0.254

*Adjusted based on general linear model for age, gender, and baseline BSCVA, endothelial plaque, depth, and mean infiltration size

**Adjusted based on general linear model for age, gender, mean infiltration size

### Secondary outcomes

**Fig. 1:**
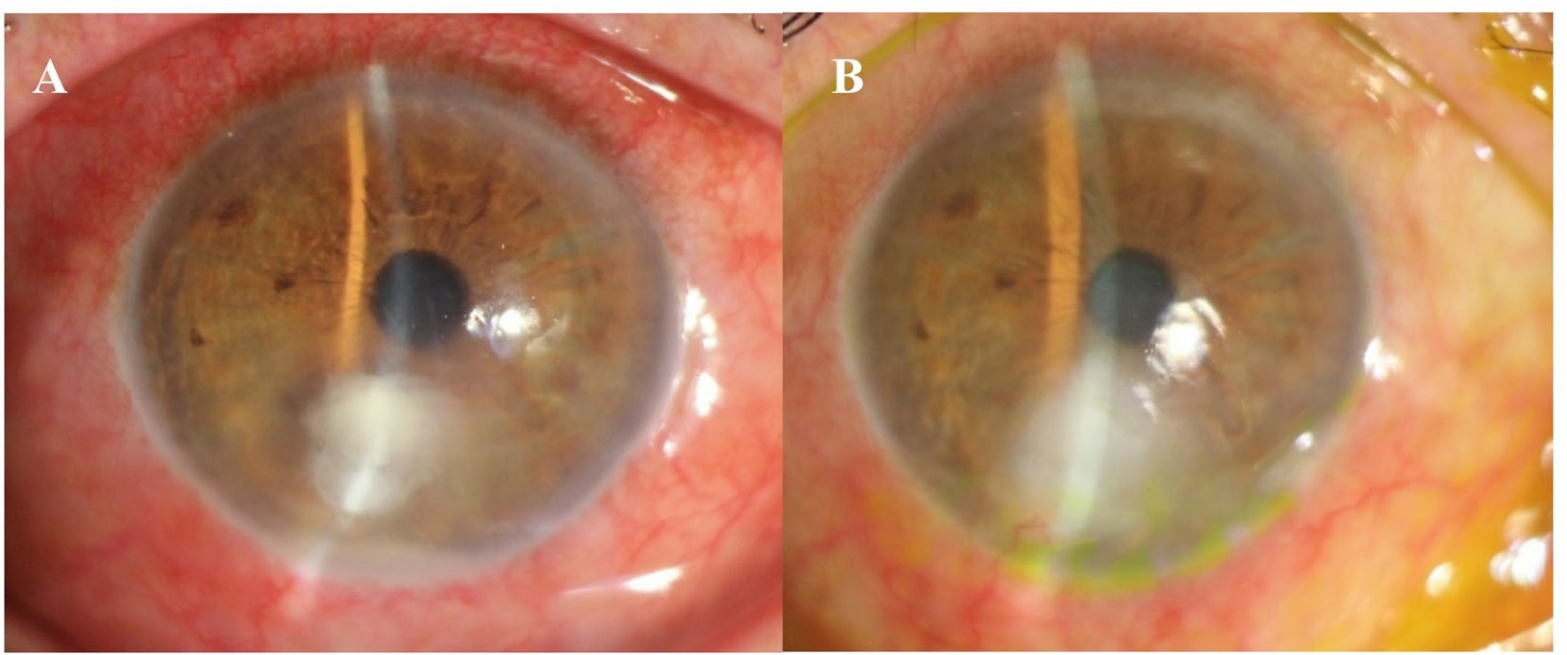
(**A**) Slit-lamp photograph shows a fungal corneal ulcer (Alternaria spp). Treated with monotherapy caspofungin0.5%, (**B**) The response to antifungal medication was noted at the last follow-up.

**Fig. 2:**
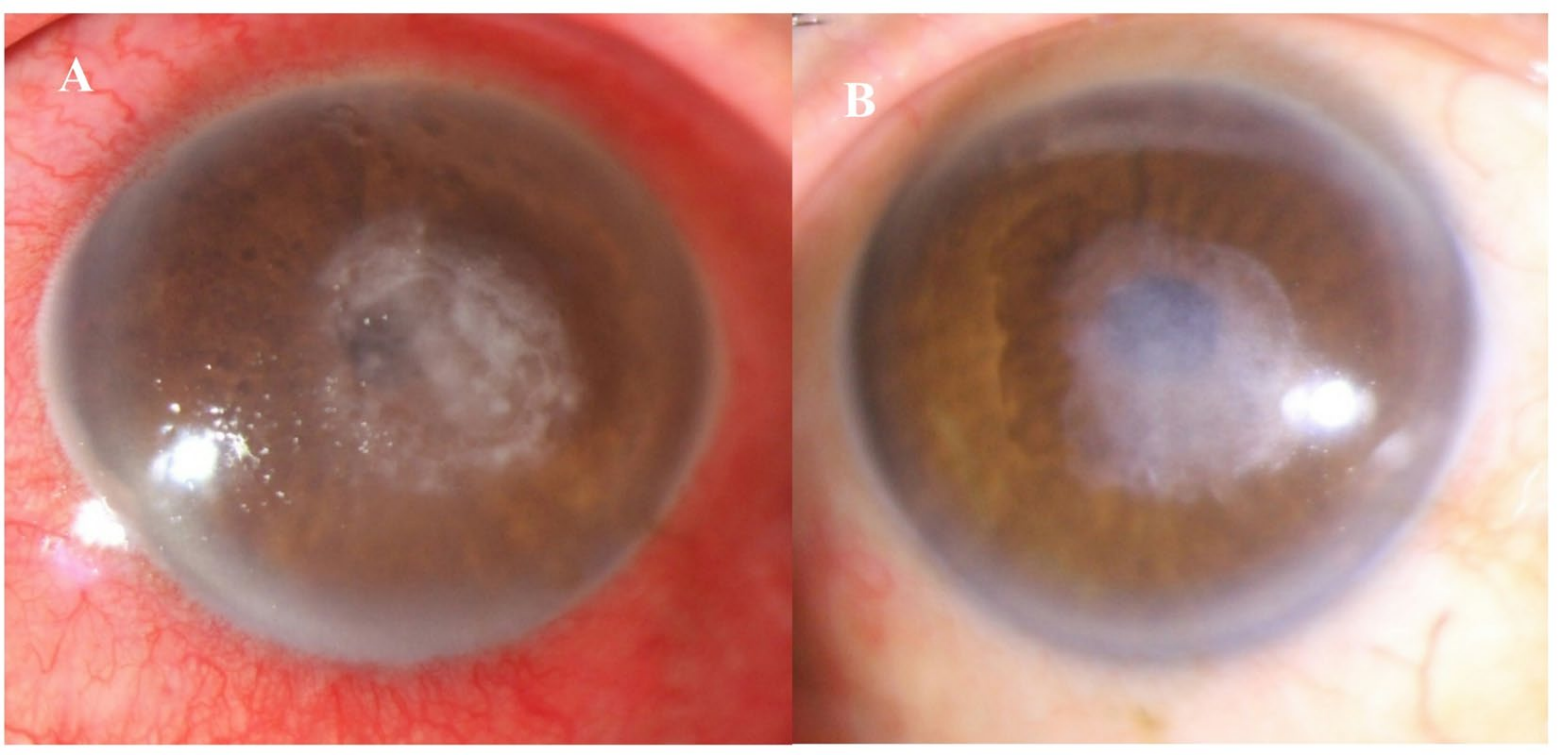
(**A**) Slit-lamp photograph reveals a 5 × 5 mm central fungal corneal ulcer. The condition was effectively treated using monotherapy with topical voriconazole 1%, (**B**) The response to the antifungal medication at the last follow-up.

The percentage of cases healed were 12 (70.6%) and 17(100%) in caspofungin and voriconazole groups, respectively (*P* = 0.060, HR,2.06 95% CI,0.97 to 4.37). The average duration for stromal infiltration healing was 45 ± 34 and 33 ± 28 day (*P* = 0.344, based on Mann-Whitney test) in the caspofungin and voriconazole groups, respectively. Figure 1 illustrates the fungal infiltration in response to topical caspofungin 0.5% treatment, while Fig. 2 shows the infiltration responding to topical voriconazole1%.

The mean size of the scar at last visit in the caspofungin group was 2.47 ± 2.1 mm and that in the voriconazole group was 3.09± 1.11 mm (*P* = 0.270).

**Fig. 3:**
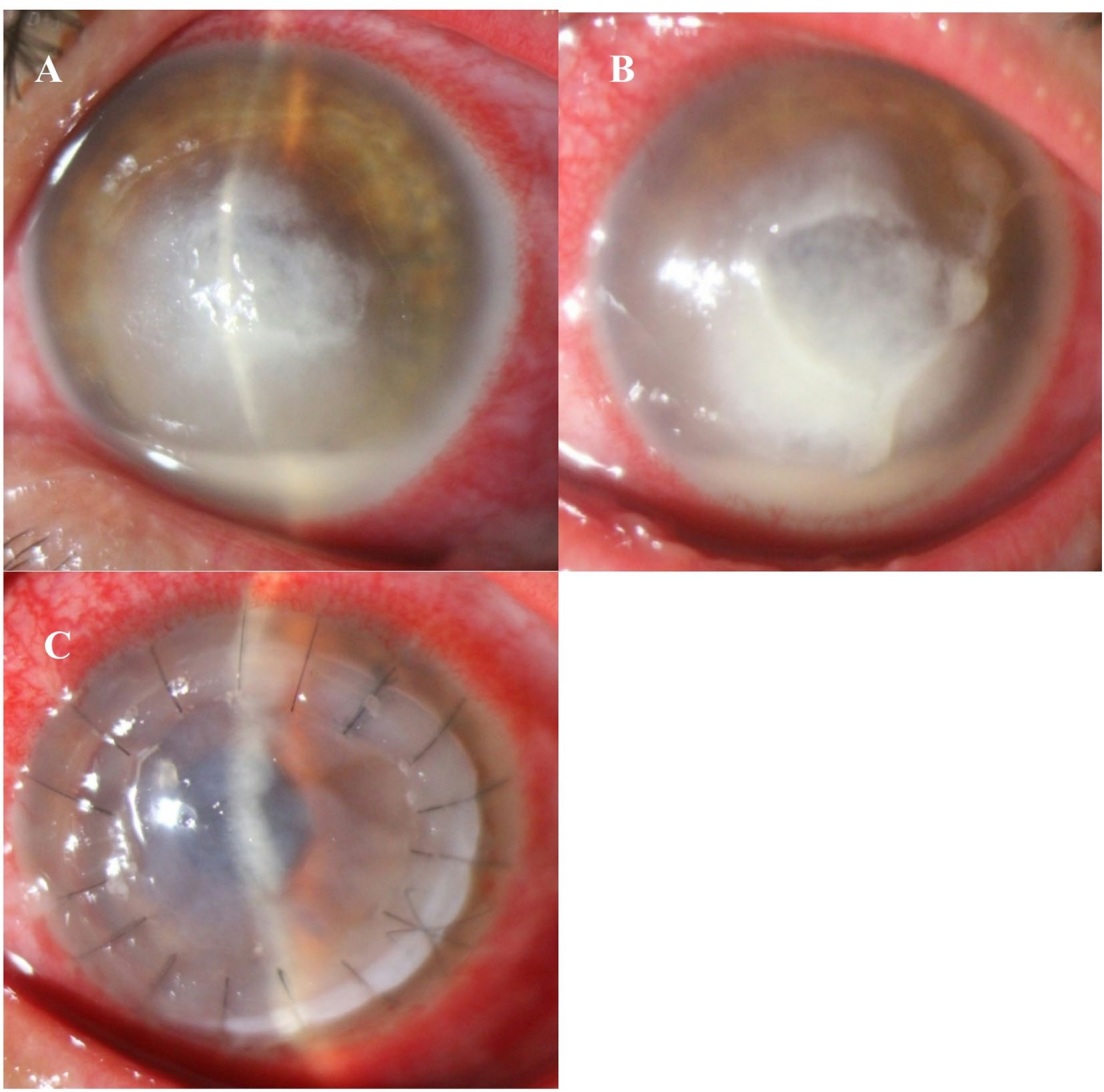
(**A**) Slit-lamp photograph reveals central fungal corneal ulcer with feathery border infiltration and hypopyon (Fusarium spp). (**B**) On the 19th day, after adding topical and systemic voriconazole to caspofungin 0.5%, the infiltration size, depth, and hypopyon progressed. (**C**) The patient underwent T-PKP on the 20th day.

During the treatment, corneal perforation occurred in 2 eyes (11.8%) in the caspofungin group and one eye (5.9%) in the voriconazole group, with no significant difference observed (*P* = 1.0). Furthermore, non-responsive infiltrations were noted in four eyes within the caspofungin treatment group, comprising three cases of Fusarium and one case of Aspergillus. (Fig. 3).  Although topical voriconazole was added for patients who failed to respond to caspofungin, none of them showed improvement with this treatment. Therefore, therapeutic penetrating keratoplasty (T-PKP) was performed. The mean size of infiltrations in the non-response to treatment that needed T-PKP was 5.07 ±1.37 mm, compared to 3.71 ±1.28 mm in those who responded successfully to treatment (*P* = 0.020) and non-response to treatment patients had greater depths of infiltration 31 ±13% (*P* = 0.103) or endothelial plaque (*P* = 0.048).

The organism was isolated in 11 patients (64.7%) in the caspofungin group and in 10 (58.8%) patients in the voriconazole group (*P* = 0.97). Among all patients the cultures media was positive (21/34;61.8% cases) and *Fusarium species* were the most common species (9/34; 26.5% cases) isolated, followed by *Aspergillus* species (6/34; 17.6% cases). (Table 3). Complete healing of stromal infiltration occurred in all 5 patients infected with Fusarium species who were treated with voriconazole 1%. In contrast, only 1 out of 4 patients in the caspofungin group demonstrated complete healing (*P* = 0.048, based on Fisher exact test). The patients in caspofungin group had large mean infiltration size 4.81± 1.60 mm, (*P* = 0.255) than 2.90± 1.60 mm in voriconazole group and deeper infiltration depth 25 ±13% than 15± 9%voriconazole group (*P* = 0.120).

**Table 3:** Fungi cultured in cases of fungal keratitis.

Organism			Total	Treatment		*P*-value	
				Caspofungin	Voriconazole		
Alternaria species			2 (5.9%)	1 (5.9%)	1 (5.9%)	1.0	
Aspergillus species			6 (17.6%)	4 (23.5%)	2 (11.8%)	0.656	
Pseudallescheria boydii			2 (5.9%)	1 (5.9%)	1 (5.9%)	1.0	
Candida species			2 (5.9%)	1 (5.9%)	1 (5.9%)	1.0	
Fusarium species			9 (26.5%)	4 (23.5%)	5 (29.4%)	> 0.99	
No growth			13(38.2%)	6(35.3%)	7(41.2%)	0.97	

## Discussion

Fungal Keratitis presents significant treatment challenges, and while natamycin 5% remains the only FDA-approved medication for this condition, alternative treatment options such as fluconazole, amphotericin B, voriconazole, and chlorhexidine are also considered^4^. Previous studies have demonstrated that topical caspofungin 0.5% is a safe and effective treatment for certain cases of FK^11,14,15^. Few studies have specifically evaluated the use of caspofungin for treating refractory fungal keratitis in humans^5,10^. This study aimed to evaluate the efficacy of topical caspofungin and voriconazole in treating FK. None of the patients experienced systemic or local toxic effects, and no patient had to discontinue caspofungin due to stinging, allergies, or corneal toxicity, which is consistent with previous studies^11,15^.

This study identified Fusarium species as the most frequent organism, followed by Aspergillus species. In Iran, Fusarium species are the primary pathogens responsible for fungal keratitis in patients^16^ in contrast to findings from Sharma et al., where Aspergillus species are more prevalent in North India^17,18^. Based on the available evidence, it is recommended to use topical natamycin 5% as the first-line treatment for Fusarium corneal ulcers, as it has demonstrated better results compared to other antifungal agents^19^. Monotherapy with voriconazole was found to be less effective than natamycin in treating Fusarium keratitis^19,20^. Due to the unavailability of natamycin in our country, voriconazole is the primary antifungal medication. Therefore, we aim to compare the efficacy of caspofungin with that of voriconazole. In the current study, complete healing of stromal infiltration occurred in all five patients infected with Fusarium species who were treated with 1% voriconazole. In contrast, only one out of four patients in the caspofungin group achieved complete healing of stromal infiltration. These results suggest that patients with Fusarium keratitis treated with caspofungin may exhibit a less favorable response, particularly those with larger lesion sizes and deeper infiltration depths. This finding aligns with our previous research^15^. Prior studies recommend natamycin as the first-line antifungal treatment for Fusarium keratitis^19,21^.

The average ulcer sizes in the two treatment groups of this study were 4.25 ± 1.54 mm and 3.74 ± 1.23 mm in caspofungin and voriconazole groups respectively. These findings are similar to those in the MUTT study, where ulcer sizes ranged from 3.8 to 4.1 mm across the different groups^22^.

Several factors, including the location of the ulcer and the extent of tissue involvement, influence the final BSCVA in cases of infectious keratitis^22,23^. In this study, we compared the BSCVA and adjusted it based on the size and depth of ulcers between the two groups of patients. It’s important to note that the difference in final BSCVA between two groups was not statistically significant. Prajna et al. compared topical natamycin and voriconazole’s effectiveness in 120 patients with fungal keratitis. Their results also indicated no significant differences in visual acuity, scar size, or the occurrence of perforations between the two treatment groups^22^.

While direct comparisons of outcomes between studies are challenging, the success rate in our current pilot study 64% and 94% in caspofungin and voriconazole groups respectively. These rates are similar to those reported for topical natamycin and voriconazole. Sharma et al. reported that 89.2% of patients treated with natamycin experienced healed ulcers, whereas only 66.6% of those treated with voriconazole had the same outcome^21^.

This study reported a corneal perforation rate of 8.8% and a need for therapeutic keratoplasty in 23.5% of cases. These findings are comparable to those reported by Sharma et al., who observed a corneal perforation rate of 6.7% and a need for T-PKP in 20.2% of cases following the early use of combination topical therapy natamycin and voriconazole^23^. In the MUTT 1 study conducted by Prajna et al., which compared topical voriconazole with natamycin, the overall rate of corneal perforation and/or the need for therapeutic keratoplasty was 16%^22^. Furthermore, in the MUTT 2 study, Prajna et al. reported a higher corneal perforation rate of 27% and a therapeutic keratoplasty requirement of 43.8% among the cases undergoing treatment than our study^24^.

In conclusion, treating fungal keratitis presents significant challenges, emphasizing the urgent need for innovative antifungal eye drop formulations. This study demonstrated that caspofungin 0.5% is as effective as voriconazole in treating fungal keratitis, and caspofungin 0.5% may be a viable primary treatment option for certain patients. However, fungal keratitis that presents with larger or deeper infiltrations or that is caused by a Fusarium infection may not respond adequately to monotherapy with topical caspofungin. This pilot study has some limitations, including the small sample size and variations in ulcer size and depth. Additionally, the unequal distribution of Fusarium species, alongside the use of voriconazole rather than natamycin—an agent demonstrated to be more effective against Fusarium species—presents further challenges for comparative analysis. Thus, the effectiveness of caspofungin compared to voriconazole for FK should be validated in larger prospective randomized controlled trials before it can be recommended as part of standard clinical practice.

## Supplementary Information

Below is the link to the electronic supplementary material.

Supplementary Material 1

## Data Availability

The datasets used and/or analyzed during the current study available from the corresponding author on reasonable request.
