# Supplementary material for: Comparative efficacy and safety of topical caspofungin 0.5% and voriconazole 1% for fungal keratitis: a pilot clinical trial
Source: Sci Rep. 2025 Nov 6;15:38879. doi: 10.1038/s41598-025-22615-w (PMC12592481; doi:10.1038/s41598-025-22615-w)

Appendix 1. Result of research based on CONSORT diagram

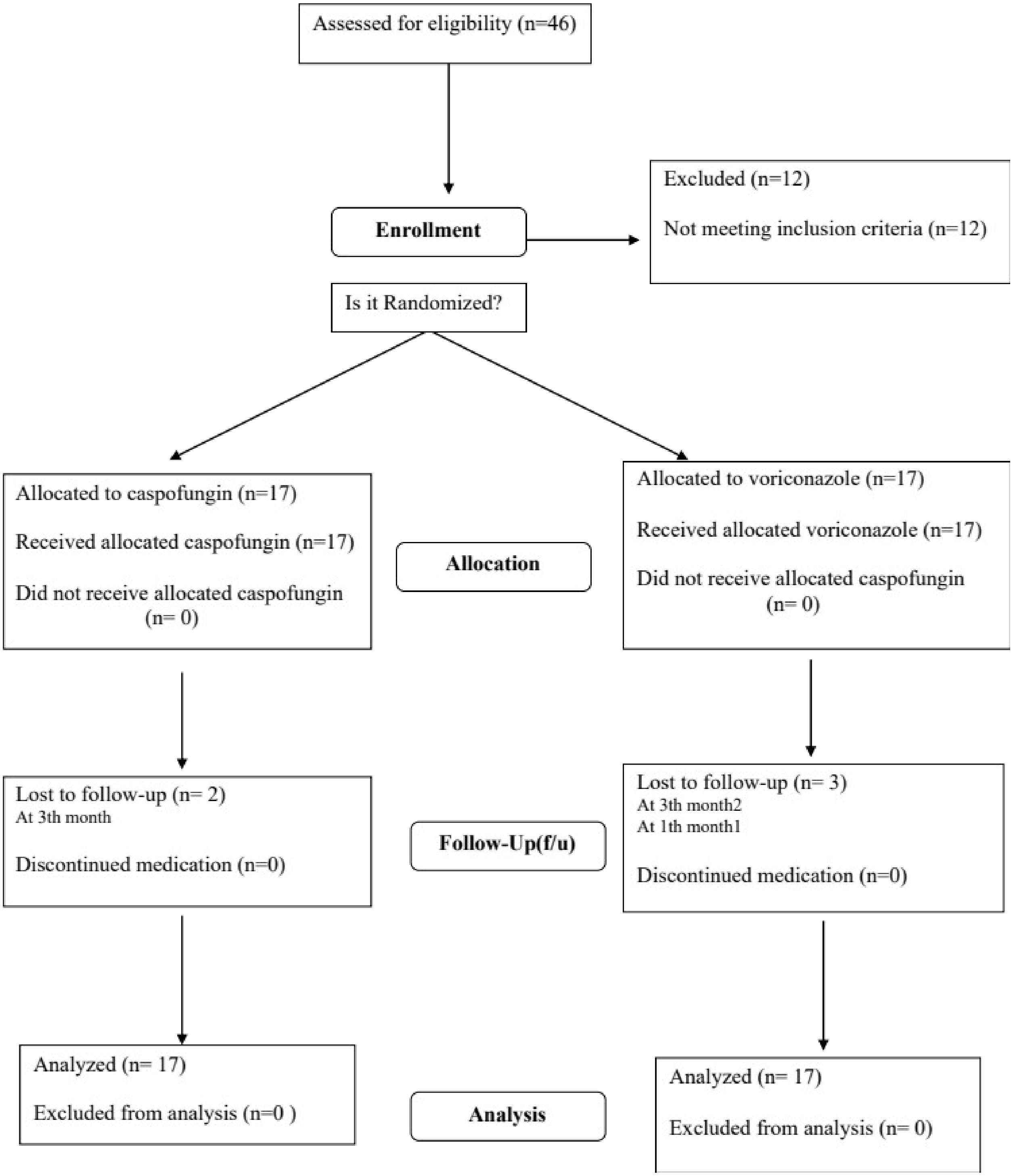

Supplement: Supplementary file 1 [file 41598_2025_22615_MOESM1_ESM.docx]
